# Boolean Network Model Predicts Knockout Mutant Phenotypes of Fission Yeast

**DOI:** 10.1371/journal.pone.0071786

**Published:** 2013-09-19

**Authors:** Maria I. Davidich, Stefan Bornholdt

**Affiliations:** Institute for Theoretical Physics, University of Bremen, Bremen, Germany; Semmelweis University, Hungary

## Abstract

Boolean networks (or: networks of switches) are extremely simple mathematical models of biochemical signaling networks. Under certain circumstances, Boolean networks, despite their simplicity, are capable of predicting dynamical activation patterns of gene regulatory networks in living cells. For example, the temporal sequence of cell cycle activation patterns in yeasts S. pombe and S. cerevisiae are faithfully reproduced by Boolean network models. An interesting question is whether this simple model class could also predict a more complex cellular phenomenology as, for example, the cell cycle dynamics under various knockout mutants instead of the wild type dynamics, only. Here we show that a Boolean network model for the cell cycle control network of yeast S. pombe correctly predicts viability of a large number of known mutants. So far this had been left to the more detailed differential equation models of the biochemical kinetics of the yeast cell cycle network and was commonly thought to be out of reach for models as simplistic as Boolean networks. The new results support our vision that Boolean networks may complement other mathematical models in systems biology to a larger extent than expected so far, and may fill a gap where simplicity of the model and a preference for an overall dynamical blueprint of cellular regulation, instead of biochemical details, are in the focus.

## Introduction

Our ignorance of the functioning of the genome, despite knowing its complete DNA sequence, illustrates the enormous role of the — far less well characterized — multitude of biochemical interactions between the genes and within the living cell. The complex web of biochemical interactions forms a computational device on which the construction, control, and maintenance of cells and organisms relies [1]. While deciphering the structure of these control networks of the living cell is a central goal of modern biology, probably the most crucial part in decrypting the full functional role of the genome is the task of reconstructing their computational dynamics with the help of mathematical models [2].

Dynamical models using the popular mathematical method of ordinary differential equations (ODE) provide for prototypical models that faithfully reproduce the dynamics of small biological regulatory networks. A prominent example is the small regulatory sub-network that controls the cell cycle in yeast [3]–[5]. ODE models are able to reproduce the complex biochemical kinetics of the central genes and proteins that make up the cell cycle control network. As an input, these models are based on the details of the biochemical interaction kinetics [6]–[8]. By construction, this results in a rather complex mathematical model, even for the relatively small yeast cell cycle network. Considering the task of constructing much larger regulatory networks in the future, it is a valid question whether, in practice, the ODE-approach will scale well to much larger networks of hundreds of nodes, or whether ODE models could be accompanied by a class of simpler models.

On a route towards simpler models, one indeed finds that ODE models sometimes capture more dynamical detail than needed for modeling certain aspects of regulatory networks. For example, when solely focusing on the sequence of biochemical activation patterns in a cell, without their exact biochemical timing, the much simpler discrete dynamical models might be sufficient. In fact, it has been observed that highly simplified network models based on Boolean (ON/OFF) states with discrete dynamics (or: networks of switches) are capable of forecasting the dynamical sequence of protein activation patterns of small regulatory networks as, for example, the cell cycle control network of yeast [9], [10].

While such Boolean network models drop the explicit representation of real time, their prediction, a temporal activation pattern sequence, represents entirely measurable properties of the biological cell as, in this case, the sequence of stages along the cell cycle [11]. Recently, in a number of systems biology applications, Boolean networks have been used to predict the dynamics across a variety of biological processes [12]. Examples range from control of development [13], [14], to signal transduction networks [15], and therapeutic target identification [16].

In this article, we study the further capabilities of a Boolean network model reproducing the temporal activation pattern sequence of a wild type regulatory network, and ask whether it is capable of predicting the dynamical phenotype of a large set of mutated networks, as well. ODE models have been shown to reproduce a considerable number of mutants for the cell cycle networks of budding yeast, fission yeast, as well as mammalian cells [17]–[19]. While one may expect that the level of detail contained in ODE models of the yeast networks is necessary in order to predict network dynamics of mutant phenotypes, also the much simpler Boolean networks can in principle predict biological states of mutated regulatory networks. Prominent examples are the cell-fate determination during Arabidopsis thaliana flower development [20], [21], as well as the effect of knockouts of transcription factors on the developmental control patterns in Drosophila melanogaster embryonal patterning (stripe formation) [22], [23]. Other studies include the mammalian cell cycle [24], a neurotransmitter signaling pathway [25], and the budding yeast cell cycle network [26].

In the following, we will focus on fission yeast and study the problem of predicting the temporal activation patterns of the cell cycle networks of mutants. *Schizosacharomyces pombe* (fission yeast) is a well characterized system, both, experimentally and theoretically, with advanced differential equation models that are particularly suited for predicting a large set of mutant phenotypes [4]. Existing contemporary ODE models [3], [5], [6], [17], [27]–[29] are able to reproduce the temporal evolution of protein concentrations along the cell cycle progression, both, for the wild type cell, as well as for a number of loss-of-function mutants, and for temperature-sensitive mutants, and over-expression mutants [30]. On this background, we here study a dynamical network model simplified to its extremes, a Boolean network, with particular attention to its ability for predicting mutant phenotypes in fission yeast. For the fission yeast wild type, the approach of a Boolean network model of the cell cycle network has been demonstrated to work well [10].

In the following we will extend this model such that it can be mutated corresponding to the subset of all known fission yeast mutants that in principle can be represented by a Boolean network. In the next section we first derive an extended Boolean network model based on the known biochemical reactions of the biological network. We then present its dynamics for the wild-type as well as for the set of known mutants. In the last section we discuss the results in comparison to experiment and to the standard ODE models of the fission yeast cell cycle.

## Methods

### The fission yeast cell cycle control network

Let us first recapitulate the main biochemical interactions of fission yeast cell cycle control, in order to subsequently construct a Boolean network representation. The fission yeast cell cycle consists of the stages G1 – S – G2 – M. In the G1 phase the cell grows. G2 is a “gap” between the main events in the cell cycle – DNA synthesis in the S phase, and separation of chromosomes followed by division into two cells in the M (mitosis) phase. After stage M, the cell enters the G1 phase again, thereby completing the cycle.

The biochemical reactions forming the control network of the fission yeast cell cycle have been studied in detail over the last years [17], [31]–[39]. The core circuit is based on the antagonist interactions of the M-promoting factor, Cdc2/Cdc13, with the inhibiting proteins Ste9, Slp1, and Rum1. Cdc2 is a cyclin dependent kinase (Cdk) and can be active only in a complex with cyclins as, e.g., Cig1, Cig2, Puc1, or Cdc13. Such post translational control forms a central part of the fission yeast network [40].

In our model, we consider the complexes Cig1/Cdc2, Cig2/Cdc2, Puc1/Cdc2, and Cdc2/Cdc13. The concentration of Cdc2 does not change during the cell cycle, however Cdc2 can exist in two states: (1) Phosphorylated on residue Tyr15, or (2) not phosphorylated. The phosphorylation of Tyr15 reduces the activity of Cdc2. For this reason, we will add an extra node to our Boolean network representation below. A new node Cdc2_Tyr15 represents the phosphorylated state of Cdc2: This node is ON if phosphorylation is removed and is OFF otherwise. The activation of Cdc2_Tyr15 together with Cdc2/Cdc13 is crucial for the G2-M transition, whereas the activation of only Cdc2/Cdc13 without Cdc2_Tyr15 corresponds to the G2 phase [4], [17]. Further, there are several helper molecules as kinase Wee1, phosphate Cdc25, and PP whose concentrations change characteristically along the cell cycle.

**Figure 1:**
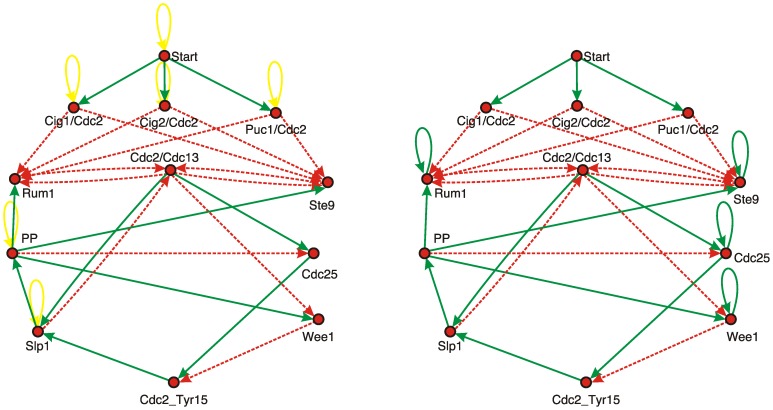
Boolean network model of the fission yeast cell cycle regulation. Nodes with states ON/OFF represent the presence of proteins. Arrows represent interactions between proteins as defined in the interaction matrix 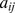 of the model (with 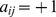 for green/solid arrows and 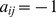 for red/dashed arrows). The dynamics is defined through a threshold function representing the switching behavior of regulatory proteins. Left: Network model with threshold function (1) and with self-degrading loops (yellow). Right: Simplified Boolean network model with threshold function as defined in eqn. (2). Both networks exhibit the same dynamical results discussed in this study. Thresholds for the nodes are chosen as described in the text. For annotations see Table 1.

A summary of all interactions between key-regulators of the fission yeast cell cycle network is given in Fig. 1 where two model versions are shown as will be discussed below. The network visualizes the interactions where proteins and complexes are represented by the network nodes, and biochemical reactions are classified into the two classes activating/inhibiting as represented by green/red links, respectively. This model is an extended version of the wild type Boolean network model for the fission yeast cell cycle [10], with a more detailed implementation of Cdc2 phosphorylation, including more Cdc2 complexes.

**Table 1:** The rules of interaction of the main elements involved in the fission yeast cell cycle regulation.

Parent node	Daughter node	Rule of activation (comments)	Rule of inhibition (comments)
Start node	Start Kinases (SK): Cdc2/Cig1, Cdc2/Cig2, Cdc2/Puc1	Start node acts as an indicator of cell mass and activates Start Kinases Cdc2/Cig1, Cdc2/Cig2, Cdc2/Puc1, +1 [28].	
SK	Ste9, Rum1		Phosphorylate, thereby inactivate, −1 [4], [28].
Cdc2/Cdc13	Cdc25	Cdc25 is phosphorylated thereby activated, +1 [28].	
Wee1, Mik1	Tyr15		Phosphorylate, inactivating, −1 [28].
Rum1	Cdc2/Cdc13		Binds and inhibits activity, −1 [28].
Cdc2/Cdc13	Rum1		Phosphorylates and thereby targets Rum1 for degradation, −1 [4], [28].
Ste9	Cdc2/Cdc13		Labels Cdc13 for degradation, −1 [4], [28].
Tyr15, Cdc2/Cdc13	Slp1	Highly activated Cdc2/Cdc13 activates Slp1, Tyr15 has to be active, too, +1 [17], [28].	
Slp1	Cdc2/Cdc13		Promotes degradation of Cdc13, thereby the activity of Cdc2/Cdc13 drops, −1 [28].
Slp1	PP	Activates, +1 [28].	
PP(Unknown phosphatase)	Ste9, Rum1, Wee1, Mik1	Activates Rum1, Ste9, and the tyrosine-modifying enzymes (Wee1, Mik1) +1 [28].	
Cdc25	Tyr15	Cdc25 reverses phosphorylation of Cdc2, thereby Tyr15 becomes active, +1 [17], [28].	
Cdc2/Cdc13	Ste9		inhibits, −1 [17].
PP	Cdc25		inhibits, −1 [28].
Cdc2/Cdc13	Wee1, Mik1		inhibits, −1 [17].

### The Boolean network model

The Boolean network model of the fission yeast cell cycle control network is defined by either one of the interaction graphs of Fig. 1, together with a set of discrete dynamical rules as defined in the following. Each node *i* in the network is assigned a binary value 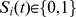, representing whether the corresponding protein is present (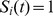) or absent (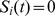) (meaning high or low concentrations and disregarding the intermediate range, as usually done in this strongly simplified picture of states just being ON or OFF). Likewise, the interactions are mapped onto discrete values as interaction strengths of 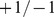, or activating/inhibiting links, as defined in Fig. 1. The update rule for the graph on the left is defined as follows. The state 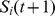 of node *i* in the next discrete time step 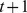 as a function of a given activation state of the network at time *t* is defined as
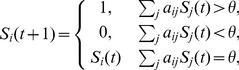
(1)with 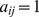 for an activating interaction (green link) from node *j* to node *i*, 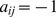 for an inhibiting (red) link from node *j* to node *i*, and 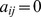 for an absent interaction. 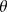 is a threshold of activation of the node *i*, which is 0 for all nodes, except three cases, Cdc2/Cdc13, Cdc2_Tyr15, and Slp1, as further explained below. This rule closely follows the approach in [9], [10]. Nodes are updated synchronously in discrete time steps. Boolean networks with such a threshold activation rule are sometimes called (Boolean) threshold networks, and are a particularly simple and elegant subset of the large class of all possible Boolean networks [41].

Cdc2/Cdc13 has a threshold of 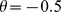, which corresponds to self-activation of the node: In the complex Cdc2/Cdc13, Cdc2 is always present, as mentioned above, and Cdc13 is constantly synthesized (there is no other positive regulation from other nodes of the system). Further, as the node Cdc2_Tyr15 is phosphorylated unless the phosphorylation is actively removed, we add a threshold rule 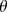 = 0.5 for this node. The third special rule is a threshold of 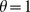 for Slp1. Slp1 is only activated by a highly active complex Cdc2/Cdc13, which corresponds to simultaneous activation of Cdc2/Cdc13 and Cdc2_Tyr15. This mechanism works as a barrier for entering mitosis. Further, as argued in [9], we add “self-degradation” (yellow loops) for those nodes that are not negatively regulated by others. This represents the degradation of proteins in the cell and is implemented as an inhibitory self-link 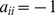.

An even simpler Boolean model has recently been formulated [42] which reduces eqn. 1 to two alternatives and does not rely on self-degrading loops:
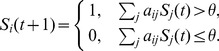
(2)The corresponding interaction graph is shown in Figure 1 on the right. In the coupling matrix here the yellow self-degrading loops are no longer necessary as the nodes now are themselves self-degrading by default (as motivated by the general biochemical degradation of all proteins). Instead, the formerly non-self-degrading nodes each acquire a self-activating coupling 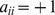. These are nodes Rum1, Ste9, Cdc25, and Wee1, except for the nodes Cdc2/Cdc13 and Cdc2_Tyr15 as they are already offset from the self-degrading threshold by their finite values of 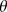. All dynamical results in this study are identically obtained by both implementations of the yeast network.

## Results

### Boolean network model for the wild type

**Figure 2:**
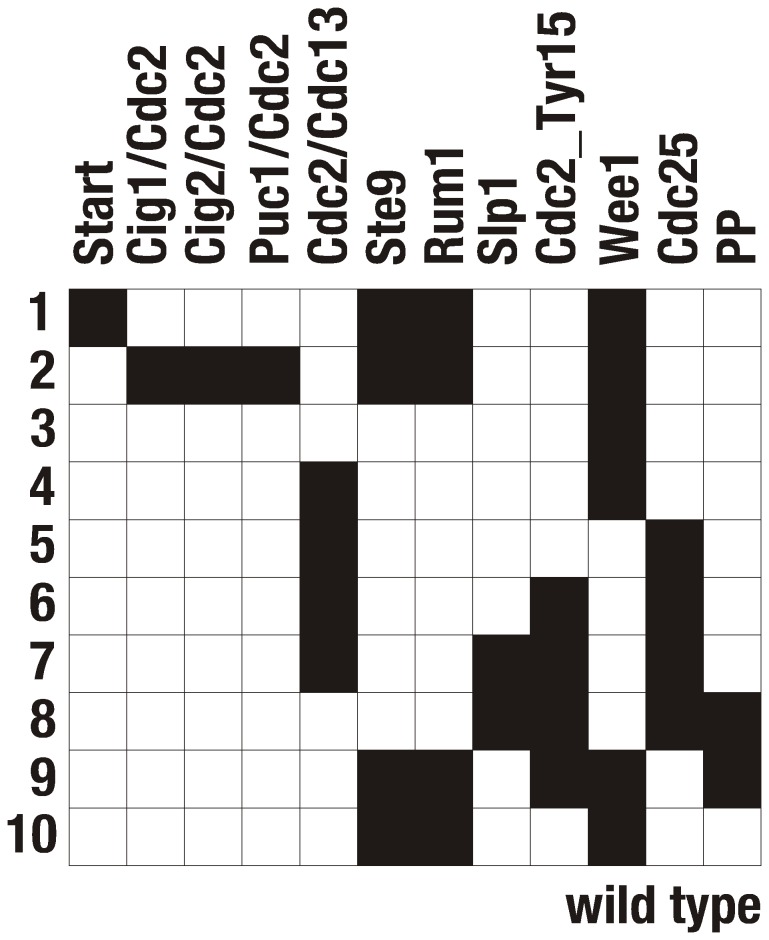
Temporal sequence of protein states of the wild-type cell cycle (time runs from top to bottom). Each column corresponds to one node in the network, each row represents one network state at a given time. The colors black/white correspond to the node's states ON/OFF (or 1/0), respectively. See Table 2 for annotation.

Let us first review the Boolean network dynamics for the fission yeast wild type cell cycle, and subsequently for different classes of mutants. The initial conditions of our model are chosen in correspondence with the biological start conditions, i.e. all nodes are in the OFF (inactive) state, except for the nodes Start, Ste9, Rum1, and Wee1 [4]. Updating the networks from this network state, initiates a sequence of network states (sets of ON/OFF states of all nodes) which reproduces the biological time sequence of protein activation during wild-type cell cycle phases G1 - S - G2 - M - G1 (see also Fig. 2). The last time step corresponds to the G1 stationary state, where the activity of all nodes is the same as at the first time step, except for the Start node which now remains OFF.

**Table 2:** Temporal evolution of protein states in the cell cycle network.

Time	Phase	Comment
1	START	Cdc2/Cdc13 dimers are inhibited, antagonists are active.
2	G1	Start kinases (SK) are becoming active.
3	G1/S	When Cdc2/Cdc13 and SK dimers switch off Rum1 and Ste9/APC, the cell passes ‘Start’ and DNA replication takes place, so Cdc2/Cdc13 starts to accumulate.
4	G2	Activity of Cdc2/Cdc13 achieves moderate level, which is enough for entering G2 phase but not mitosis, since Wee1/Mik1 inhibits residue of Cdc2_Tyr15 that does not allow total activation.
5	G2	With moderate activity Cdc2/Cdc13 activates Cdc25.
6	G2/M	Cdc25 reverses phosphorylation, removing the inhibiting phosphate group and activating residue of Cdc2_Tyr15.
7	G2/M	Cdc2/Cdc13 reaches high activity (Cdc2/Cdc13 and Tyr15 are both active) sufficient to activate Slp1/APC and the cell enters mitosis.
8	M	Slp1 degrades Cdc13 and activates unknown phosphatase.
9	M	Antagonists of Cdc2/Cdc13 are reset.
10	G1	Cdc2 becomes inactive as Cdc13 is degraded, cell reaches G1 stationary state.

**Figure 3:**
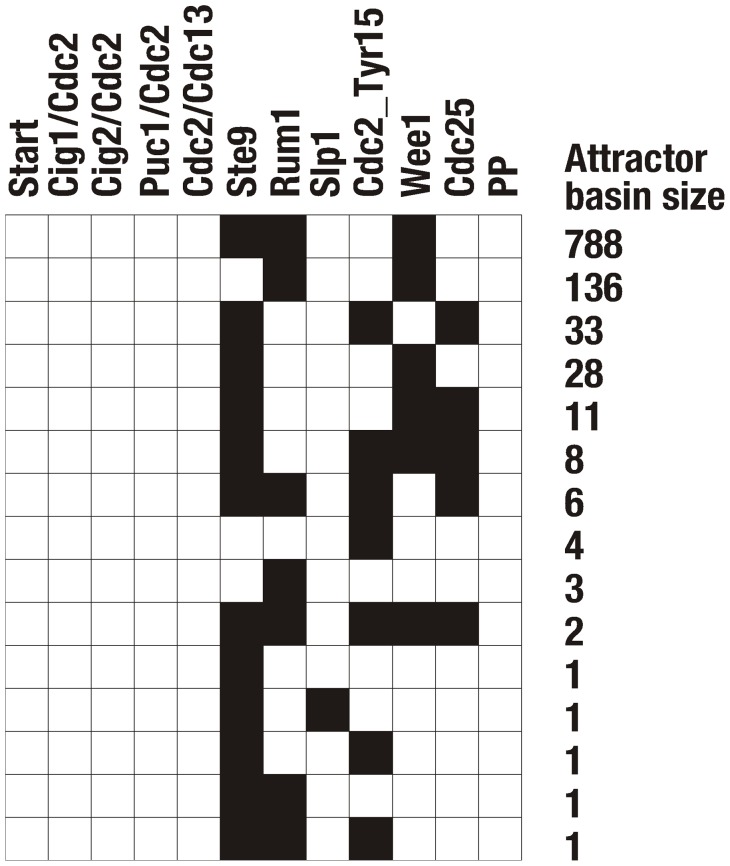
Attractors of the Boolean network model of the wild-type fission yeast cell cycle network, as described in Fig. 1. Each column is associated with a node in the model, each row represents an attractor state (fixed point of the dynamics). The basin size of each fixed point is given by the number of different initial states that converge onto this fixed point.

When running the model starting from each one of the 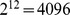 possible initial states, we obtain an overview of the state space of the Boolean network. One observes that each initial state flows into one of only 15 stationary states (fixed points), as summarized in Fig. 3. The largest attractor belongs to a fixed point attracting 77% of all network states. Our first observation is that this fixed point exactly coincides with the biological G1 stationary state (see Fig. 3) of the cell. Thus, the biological target state is the dominant attractor of the network dynamics. As soon as the system reaches this state with the specific corresponding combination of active and inactive proteins, it remains there.

### Mutations

Three prominent types of mutations which are well applicable to the yeast cell are temperature-sensitive, loss-of-function, and over-expression mutations. Temperature-sensitive mutants largely correspond to reduced activity in protein production, loss-of-function mutants to zero-activity of certain nodes, and over-expression mutants to an increased activity of a protein. In differential equation models, for modeling the temperature-sensitive mutants the appropriate kinetic constants are reduced e.g. by a factor of 10% [4], [17], [28]. Similarly, for loss-of-function mutants these constants are set to zero, whereas for over-expression mutants they are increased by a factor of two or more.

Not all of these mutants can be represented in the framework of a Boolean network model of the cell cycle control network. In particular there is no straightforward mapping for temperature-sensitive mutations (in the following denoted by superscript 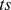), where the activity of proteins changes slightly. For this reason we mostly model loss-of-function and over-expression mutations. In the case of loss-of-function mutations, for example, the mapping is obvious and involves simply setting the corresponding nodes to the inactive state permanently. In the following we describe the dynamical properties and biological interpretation of all mutations that have been modeled with the Boolean network model.

#### Wee1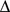 and Cdc25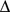 mutants

**Figure 4:**
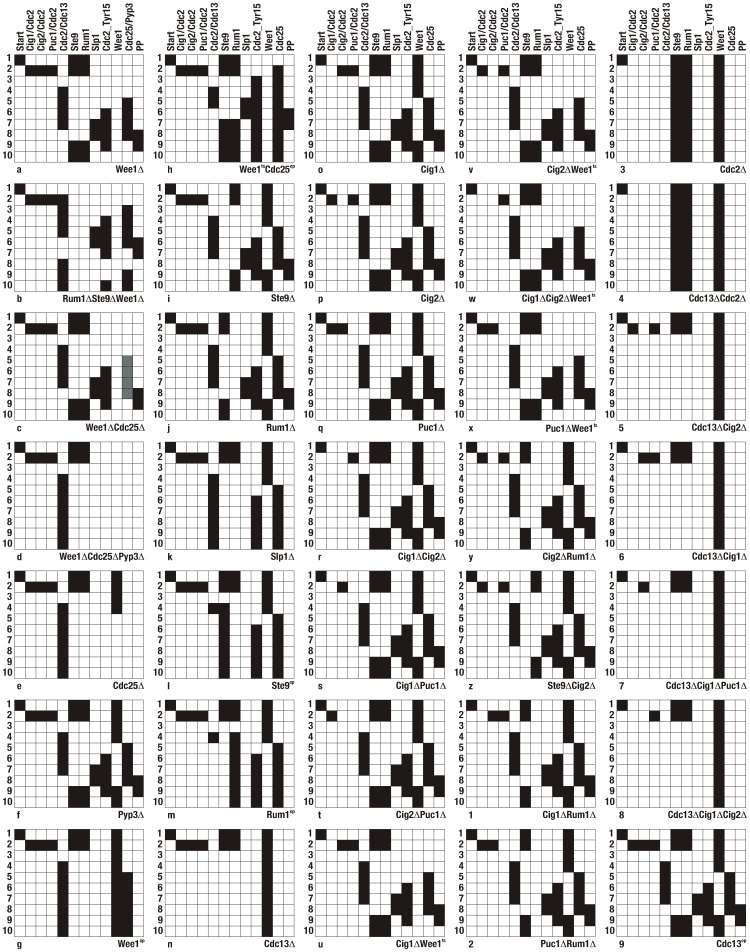
Mutant phenotypes: Temporal evolution of protein states for each mutant phenotype (time runs from top to bottom). Each column corresponds to one node in the network, each row represents one network state at a given time. The colors black/white correspond to the node's states ON/OFF (or 1/0), respectively (grey for Pyp3). See Table 3 and text for details.

The duration of the S and G2 phases are controlled by down-regulation of Wee1 by Cdc2/Cdc13. If Wee1 is absent (denoted as Wee1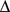), then the cell enters mitosis with a smaller size, but it stays viable [43]. In the Boolean model, implementation of the Wee1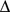 non-function (or knockout) mutation is straightforward. The temporal sequence of protein activation states here is the same as in the wild-type model. The system has one fixed point which corresponds to the G1 stable state (Fig. 4.a).

**Table 3:** Fission Yeast mutant phenotypes represented by the Boolean network model.

Strain	Deleted node(s)	Model	Real
WT	none	G1	V
Wee1Δ	Wee1	G1	V
Rum1Δ Ste9Δ Wee1Δ	Ste9, Rum1, Wee1	OSC	L
Wee1Δ Cdc25Δ	Wee1; Cdc25	G1	V
Wee1ΔCdc25ΔPyp3Δ	Wee1, Cdc25, Pyp3	G2	G2, L
Wee1*^op^*	*θ* = −0.5,*a_ii_* = 1	G2	G2, L
Cdc25Δ	Cdc25	G2	G2, L
Pyp3Δ	Pyp3	G1	V
Wee1*^ts^*Cdc25*^op^*	Wee1,*θ* = −0.5,*a_ii_* = 1	G1 (−G2)	L
Ste9Δ	Ste9	G1	V
Rum1Δ	Rum1	G1	V
Rum1*^op^*	*θ* = −0.5,*a_ii_* = 1	G2	G2, L
Ste9*^op^*	*θ* = −0.5,*a_ii_* = 1	G2	ER, L
Slp1Δ	Slp1	G2-M	M, L
Cdc13Δ	Cdc13	G1-S	ER, L
Cig1Δ	Cig1	G1	V
Cig2Δ	Cig2	G1	V
Puc1Δ	Puc1	G1	V
Cig1ΔCig2Δ	Cig1, Cig2	G1	V
Cig1ΔPuc1Δ	Cig1, Puc1	G1	V
Cig2ΔPuc1Δ	Cig2, Puc1	G1	V
Cig1ΔWee1*^ts^*	Cig1, Wee1	G1	V
Cig1ΔCig2Δ Wee1*^ts^*	Cig1, Cig2, Wee1	G1	V
Cig2ΔWee1*^ts^*	Cig2, Wee1	G1	V
Puc1ΔWee1*^ts^*	Puc1, Wee1	G1	V
Cig2ΔRum1Δ	Cig2, Rum1	G1	V
Ste9ΔCig2Δ	Ste9, Cig2	G1	V
Cdc2Δ	Cdc2	G1-S	L
Cdc13ΔCig1Δ	Cdc13, Cig1	G1-S	ER, L
Cdc13ΔCig2Δ	Cdc13, Cig2	G1-S	G1, L
Cdc13ΔCig1ΔPuc1Δ	Cdc13, Cig1, Puc1	G1-S	ER, L
Cdc13*^op^*	−1<*θ*<−0.5	G1	V
Cdc13ΔCig2ΔCig1Δ	Cdc13, Cig2, Cig1	G1-S	L

The wild type (WT) is listed for comparison. For each mutant, the modeling details are given (deleted nodes, thresholds),as well as the dynamical outcome (fixed point or OSC for oscillation). For comparison, the experimental viability/lethality (V/L) of the real fission yeast cell for the respective mutations is given. For further details see text and Fig. 4.

However, if some other antagonist of Cdc2/Cdc13 is also mutated, as in the mutants Rum1
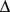
Wee1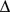 or Ste9
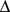
Wee1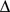, then the cell divides too fast and does not have enough time to grow [28]. With every division, the cells get smaller and smaller until they are not viable any more. In our model, start kinases Cig1/Cdc2, Cig2/Cdc2, and Puc1/Cdc2 are not influenced by Rum1 and Ste9 for simplicity. In fact, Cig2 is partly inhibited by Rum1 and possibly by Ste9 [17], [28]. For this reason one cannot separate Wee1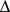 and Rum1
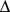
Wee1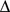, Ste9
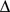
Wee1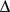 mutations in our Boolean network model. On the other hand, the model reproduces the triple mutation Rum1
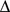
Ste9
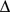
Wee1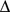 (Fig. 4.b). In this case the system shows oscillations and is not viable. As shown in Fig. 4.b, step 10 repeats step 4, such that the system goes through the same sequence of states, periodically.

In order to model the Wee1
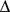
Cdc25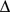 mutation, one has to take into account that Cdc25 has a backup enzyme, called Pyp3. Pyp3 is a tyrosine-phosphate with a much lower activity, which means that Cdc2 is only weakly de-phosphorylated, when Pyp3 is present. Therefore, the Wee1
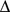
Cdc25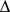 mutation is modeled as follows: Node Wee1 is deleted and the link connecting Cdc25 to Cdc2_Tyr15 is set to a lower value. We choose a lower weight of 0.75 instead of the usual weight 1.0 (the discrete dynamical model is insensitive to the exact value of the weight smaller than 1.0 and larger than 0.5). The simulation of this mutant network lets the cell cycle go through all phases with the temporal pattern of proteins as in the wild type network except for Wee1 being inactive in this mutant (Fig. 4.c). This is consistent with experimental data [28]. The removal of the nodes Cdc25 and Wee1 corresponds to a triple mutant Wee1
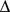
Cdc25
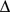
Pyp3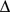, when Tyr15 stays phosphorylated. This mutation is not viable. The cell cannot enter mitosis, since Tyr15 stays phosphorylated, thereby preventing Cdc2/Cdc13 to reach high activity. The model reproduces this as can be seen in Fig. 4.d where Cdc2_Tyr15 stays inactive and the cell cycle does not enter the mitosis sequence. Another non-viable mutant is Cdc25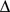 caused by the back-up enzyme Pyp3 being weaker than Wee1. In the model, the cycle remains in the G2 phase indefinitely (Fig. 4.e) in agreement with experimental results [28]. The mutant Pyp3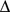 (modeled again through a lowered weight of the link connecting Cdc25 to Cdc2_Tyr15) is similar to the wild-type [44] (Fig. 4.f).

Let us now consider modeling overexpression of proteins and how they could be realized in a Boolean network model. An overexpression mutant (*op*) has the activity (or concentration) of one or more proteins significantly increased. Within the framework of our model, overexpression is interpreted as equivalent to the effect of a small constant positive input, which corresponds to a negative threshold 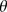 and a self-activating link 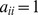 in equation (1). In particular, moderate overexpression is represented by a negative threshold 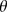 in the model, only, and an additional self-activating link is set in the case of high overexpression. Here and further we choose 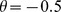 for all over-expressed mutants (without loss of generality, as any negative value larger than −1 has the same effect on the discrete threshold function of the node). Slight overexpression of Wee1 (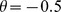) suggests that the cell stays viable [31]. However, if the level of overexpression of Wee1 increases, the model cell cycle is blocked in the G2 phase. This is in accordance with [43] (Fig. 4.g).

Finally, modeling temperature-sensitive (*ts*) mutants as in Wee1*^ts^* Cdc25*^op^* in the Boolean framework needs a second thought since Boolean variables in the model do not distinguish between reduced activity and non-activity. Therefore we substitute mutation Wee1*^ts^* with Wee1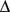 and, for the overexpression (or overproduction) mutant Cdc25*^op^*, reduce the threshold of activation to 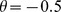 and set 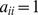. In Fig. 4.h one sees that mitosis happens very quickly without an appropriate G2 phase, consistent with mitosis being initiated before the replication of DNA is completed.

#### Mutations of Cdc2/Cdc13 antagonists: Ste9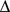, Rum1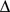, Slp1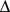 mutants

Fission yeast survives in the absence of Ste9 or Rum1 [45] and grows normally. The temporal protein evolution in the Boolean network model is consistent with this fact (Fig. 4.i,j). The system has one fixed point G1 that is reached after the evolution through all cell cycle phases G1-S-G2-M. However, the absence of the other Cdc2/Cdc13 antagonist, Slp1, has a lethal effect. Recent studies [46] demonstrate that Slp1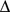 is a nonviable mutation which prevents mitosis. The dynamical behavior of the model for Slp1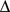 shows that the system reaches a fixed point, which corresponds to the late G2 phase, right before entering mitosis. The evolution of the proteins initially coincides with the wild type pattern sequence, but then freezes at step 6 (Fig. 4.k).

High overexpression of Ste9 is observed to prevent mitosis via endoreplication, leaving the cell nonviable [43]. Our model is able to reproduce that Ste9*^op^* prevents mitosis [47], however, does not represent the full phenomenology of endoreplication (Fig. 4.l). Finally, it is interesting to look at different levels of overexpression in the other antagonist of Cdc2/Cdc13, Rum1. Experiments find that a mild level of Rum1 overexpression (4-fold compared to wild-type), does not interfere with the mitotic cycle. However, when the level of expression is further increased (8-fold over wild-type) the cell is blocked in the G2 phase [43]. The Boolean network model finds a similar pattern: For moderate overexpression of Rum1 (represented as an activation threshold 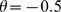) the Boolean network model exhibits wild-type behavior, however, when the level of expression is further increased (represented as 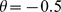 plus a self-activating link 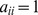), the model dynamics is blocked in G2 (Fig. 4.m).

#### Mutations of cyclins: Cig1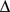, Cig2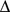, Puc1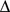, Cdc2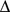, and cyclin-dependent kinase Cdc13
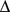


The essential cyclin for the fission yeast cell cycle is Cdc13. The presence of Cdc13 is vital for normal progression through the cell cycle [28]. In the absence of Cdc13, the cell elongates abnormally and undergoes endoreplication instead of entering the M phase. The model predicts lethality for the mutant Cdc13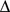 and that the cell cycle does not enter mitosis. The start kinases Cig1/Cdc2, Cig2/Cdc2, and Puc1/Cdc2 switch off the Cdc2/Cdc13 antagonists during the G1-S phases, but in the absence of Cdc13 the cell cycle cannot evolve further. The system remains on the fourth step of the wild-type cell cycle evolution (Fig. 4.n).

Start kinases Cig1/Cdc2, Cig2/Cdc2, and Puc1/Cdc2 are responsible for deactivation of Cdc2/Cdc13 antagonists. Mutations of cyclins of the start kinase only affect the duration of the G1 phase, extending its duration. Thereby mutants Cig1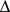, Cig2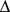, Puc1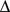, as well as their double mutants and triple mutants Cig1
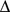
Cig2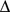, Cig1
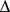
Puc1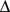, Cig2
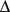
Puc1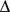, and Cig1
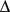
Cig2
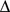
Puc1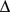 are viable. Due to simplifications of the start kinase interactions we made in the model, it is able to reproduce only single and double mutations, but not triple mutations (see Figs. 4.o,p,q,v,r,s,t). Due to the fact that the time in the Boolean model is discrete, one cannot distinguish the start kinase mutants from the (discrete) temporal pattern of the wildtype. In fact, the temporal evolution is similar to the wildtype. Double mutations and triple mutations Cig1
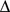
Wee1*^ts^*, Cig2
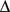
Wee1*^ts^*, Cig1
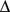
Cig 2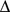 Wee1*^ts^*, Puc1
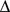
Wee1*^ts^*, Cig2
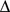
Rum1, and Ste9
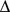
Cig2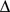 exhibit intact cell cycle dynamics in the Boolean network model, as well (Fig. 4.u,v,w,x,y,z). The model further predicts the double mutant Cig1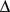 Rum1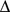 to be viable (Fig. 4.1). Experimentally it is known that the triple mutation Cig1
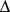
Cig2
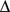
Rum1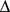 is viable [48] from which we conclude that the model result is realistic. Also, the model predicts mutation Puc1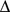 Rum1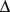 to be viable (Fig. 4.2). The knock-out mutation Cdc2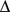 is known to be lethal [49], and in the Boolean model is predicted to block the cell cycle in G1 (Fig. 4.3). Our model further suggests that the double mutation Cdc2
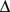
Cdc13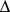 is lethal (Fig. 4.4). For the double mutant Cdc13
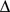
Cig2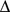 the model cell cycle is blocked in G1 (Fig. 4.5), in accordance with experiment [43]. The other double mutant Cdc13
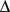
Cig1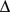, as well as the triple mutant Cdc13
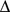
Cig1
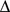
Puc1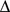 are known to enter endoreplication and are not viable [50]. Similar to Cdc13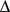 the model reproduces lethality without representing the details of endoreplication (Figs. 4.6, 4.7). Cells with the triple mutation Cig1
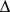
Cig2
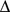
Cdc13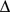 are known to be arrested before replication [51] as observed in the model as well (Fig. 4.8). Finally, under moderate overexpression of Cdc13 (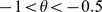, where 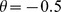 is the default threshold of Cdc13), the cell remains viable (Fig. 4.9). However, a large increase of Cdc13 activity increases the speed of the cell cycle, the cell cycle gets too short such that the cells cannot complete DNA replication, which creates a cut phenotype. Such a phenomenology is beyond what a Boolean network model can represent in detail, as the model explicitly simplifies the time axis.

## Discussion

Let us briefly discuss the dynamics of the Boolean network model and how it compares to ODE models of mutated yeast.

### Prediction of mutants

Two types of mutants turned out to be particularly suited for translation into the Boolean network model framework. The loss-of-function mutations were implemented by deleting the corresponding node(s). All loss-of-function mutations were reproduced in the model except a small number, i.e. Rum1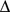 Wee1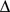, Ste9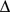 Rum1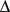, Ste9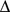 Wee1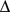, Cig1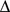 Cig2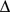 Puc1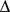. We note that these belong to a sector where the Boolean network model makes dramatic simplifications in the interactions between some proteins.

For overexpressed mutations an additional constant positive input, and for highly overexpressed mutations a self-activating link have been added to the activation rule. Also here, the successfully reproduced overexpressed mutants do not cover all known mutants, e.g. Cdc25*^op^* is not reproduced. Here, the discrete representation limits the model by the mapping of intermediate activation states to Boolean 0/1 states.

In spite of the considerable simplification of the discrete representations of continuous protein concentrations as well as of continuous interaction strengths, the Boolean network model correctly classifies viability/lethality of 32 mutants, which corresponds to about three quarters of all known mutants for the given set of involved proteins. The remaining set of mutants containing Wee1*^ts^*, Cdc25*^ts^*, Wee1*^ts^* Cdc25*^ts^*, Ste9
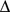
Rum1
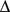
Wee1*^ts^*, Slp1*^ts^*, Pyp3*^op^*, Cig2*^op^*, Cdc25*^op^*, Rum1
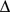
Wee1*^ts^*, and Ste9
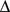
Wee1*^ts^* is not reproducible and shows the clear limitations of the Boolean discretization in the model. Representation of temperature-sensitive and over-expression mutants does not find a detailed implementation in this framework.

In particular, one cannot represent temperature-sensitive mutants properly, for example, as 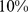 reduced activity. Further, the mutant Wee1
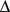
Rum1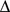 does not find its expression in a Boolean-discrete-time-step framework, since this mutant's effect is an accelerated division speed. The mutations Ste9
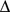
Rum1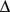 and Cig1
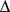
Cig2
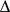
Puc1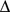 are not reproducible due to the particular simplifications of the interaction structure in the model. On the other hand the model reproduces a number of mutants which were not modeled before: Cig1
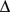
Puc1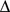, Cig2
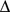
Puc1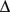, Cdc2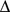, Cig1
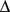
Cig2
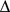
Cdc13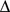, Cdc13
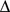
Puc1
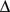
Cig1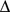.

As an overall picture, the Boolean network model appears to represent a blueprint of the cell cycle control dynamics which not only covers the wild type protein patterns sequence, but also the dynamical activation patterns of a considerable number of mutants. The fact that this variety of phenomenology is represented solely on the basis of the interaction topology underlines the general observation of the particular importance of network structure in regulatory networks [5], [22].

### Comparison to ODE model prediction of mutants

It is interesting to compare the minimalistic Boolean model with existing ODE models for the fission yeast cell cycle [3], [6], [17], [27], [28], [43]. Firstly, it is important to remark that at the time of writing there is no general ODE model for the fission yeast cell cycle that would cover all known details of the process at the same time. During the last decade, a series of different mathematical models were constructed where each of them concentrates on certain aspects of the process. Existing ODE models are tested against a steadily increasing number of mutants for fission yeast, from a set of 22 mutations earlier [30], and extending a current standard set of 42 mutants (with more target proteins) even further by exploring other mutation types [43].

Going through different versions of models one notices many similarities between the ODE models and our Boolean model. Firstly, starting with initial conditions as in [3], [17], [27], [28], the system evolves through the same sequence of states. The second evidence is the robustness of the models to the initial conditions: Our Boolean model has a dominant attractor, attracting most of the trajectories starting from different initial conditions [17]. The third evidence is the similarity in dynamical properties of mutations. In particular, the following mutations: Rum1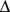, Ste9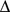, Wee1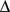, Cig1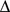, Cig2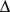, Puc1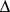, Cig1
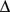
Cig2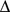, Cig2
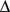
Ste9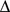, Wee1
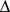
Cdc25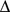, Cig1
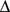
Wee1*^ts^*, Cig1
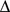
Cig2
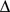
Wee1*^ts^*, Cig2
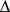
Wee1*^ts^*, Cig2
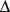
Rum1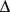, Pyp3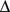 are predicted to be viable in both approaches [3], [17], [27], [28], which is confirmed by experimental data. Non-viable mutations, such as Cdc13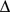, Cdc13
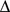
Cig1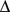, Cdc13
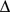
Cig2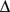, Rum1
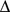
Ste9
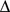
Wee1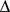, Cdc25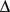, Slp1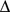, Ste9*^op^*, Wee1*^op^*Cdc25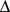, Wee1
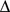
Cdc25
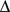
Pyp3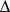 are blocked in the same phases in both model approaches. It is interesting to note that the mutant Rum1
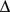
Ste9
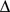
Wee1*^ts^* is predicted to be not viable in ODE models [30] since cells are considered to be too small to be viable. In the Boolean network model one observes a similar result: The discrete dynamical model ends up in a limit cycle which corresponds to the situation where a cell passes through phases too quickly without waiting for a cell mass signal. Finally, in addition to these previously modeled mutants, some of the mutations reproduced by the Boolean model above have not been modeled with the ODE approach so far.

## Conclusion

As a main result, our current Boolean network model reproduces the major results of the ODE models concerning the viability of various fission yeast mutants, except for the temperature-sensitive and some overexpression mutations which cannot be translated to the Boolean network formalism. Further our model reproduces the main general observations of the ODE model as the robustness to initial conditions and the sequence of dynamical states for the majority of the mutations. Remarkably, these results are obtained despite the lack of continuous time in the Boolean model: We explicitly drop the prediction of real time in the model, and solely consider the discrete temporal sequences of activation states of proteins.

Why does the Boolean network model approach work so well, despite the significant simplifications as compared to ODE models? The relationship between continuous and Boolean models has been discussed in detail [52], and in particular it has been shown that a Boolean network can be constructed from an existing ODE model in a mathematically well defined manner and with well defined limits on its validity [53]. From another perspective, there are indications that often biological molecular networks are so robustly designed [54]–[56] that timing is not a critical factor, and one can drop accurate reproduction of time for the sequence of states as, e.g. demonstrated for S. cerevisiae [57]. Finally, for mutants of S. cerevisiae, a Boolean network study [26] traces robustness against mutations to specific sub-networks of the budding yeast cell cycle network. Here, the exact timing of events and the order of the nodes' updates does not have a large impact on the fundamental behavior of the system, s.t. the synchronous update scheme is justified as a suitable approximation. In short, it seems that, with these biological systems, we are modeling enormously robust dynamical networks which, vice versa, allow for enormous simplifications in their dynamical models.
